# Single-chamber atrial pacing + Micra AV implantation: A case report

**DOI:** 10.1097/MD.0000000000039718

**Published:** 2024-09-20

**Authors:** Yongxiang Cai, Mingjiang Liu, Jianhong Tao, Yijia Tang, Yan Xiong

**Affiliations:** a Department of Cardiology, Sichuan Academy of Medical Sciences & Sichuan Provincial People’s Hospital, Chengdu, Sichuan, China; b School of Medicine, University of Electronic Science and Technology of China, Chengdu, Sichuan, China; c Institute of Cardiovascular Disease & Department of Cardiology, Sichuan Provincial People’s Hospital, School of Medicine, University of Electronic Science and Technology of China, Chengdu, Sichuan, China.

**Keywords:** atrioventricular synchronization, Ebstein anomaly, leadless pacing, Micra AV

## Abstract

**Rationale:**

The use of transvenous pacing leads is associated with the risk of tricuspid valve dysfunction, mainly due to the continuous presence of the leads can have an impact on subsequent tricuspid function and possible operation injury of the tricuspid valve during implantation or operation.

**Patient concerns:**

A 69-year-old female with a history of syncope for 9 months was admitted to the hospital. The electrocardiogram showed sinus bradycardia, junctional escape rhythm, and a heart rate of 44 bpm. Echocardiography suggested a downward displacement and severe insufficiency of the tricuspid valve and atrial septal defect.

**Diagnoses:**

The cause of syncope was considered to be sick sinus syndrome. The patient was diagnosed with Ebstein anomaly and is considered a candidate for surgical intervention.

**Interventions:**

To avoid aggravating tricuspid insufficiency by pacing leads crossing the tricuspid valve and hindering subsequent tricuspid valve surgery, a single-chamber pacing mode with atrial pacing (AAI) lead and Micra AV was chosen for maintaining atrioventricular synchrony after multidisciplinary discussion.

**Outcomes:**

The patient had stable parameters and was in good general condition at 1- and 3-month outpatient follow-ups after discharge.

**Lessons:**

This is the first case of new implantation of single-chamber atrial pacing + leadless ventricular pacing with Micra AV, an alternative strategy to epicardial or coronary sinus system for tricuspid valve displacement and severe tricuspid regurgitation.

## 1. Introduction

Lead-induced tricuspid regurgitation (TR) is 1 of the complications after the implantation of a permanent pacemaker, accounting for 25% of all TRs.^[1]^ Studies have shown that lead-induced TR can increase both short and long-term mortality rates in patients.^[2]^ Ebstein anomaly (EA) is a rare and complex congenital heart disease characterized by a downward displacement of the tricuspid valve, affecting 0.39 to 0.72 per 10,000 newborns.^[3]^ EA is caused by abnormal development of the tricuspid valve, leading to a series of related structural abnormalities. Approximately two-thirds of EA patients die from heart failure. Surgical treatment is recommended as an effective treatment for symptomatic or congestive heart failure patients with severe tricuspid valve insufficiency.^[4]^ In this context, to avoid the pacing lead passing through the tricuspid valve, this paper described a new pacing procedure to maintain atrioventricular sequential pacing for the treatment of patients with EA and sick sinus syndrome (SSS).

## 2. Case presentation

**Figure 1.**
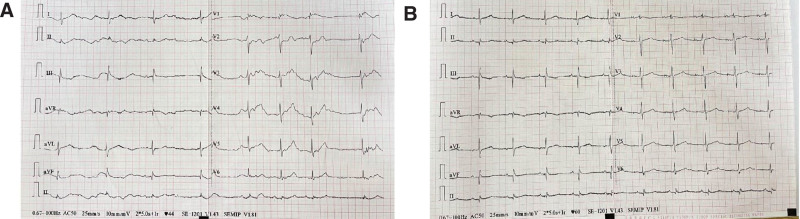
Preoperative and postoperative electrocardiogram of the patient. (A) Admission electrocardiogram (sinus bradycardia, 44 bpm, junctional escape rhythm). (B) Postoperative electrocardiogram (atrial 60 bpm pacing, ventricular self-down transmission).

**Figure 2.**
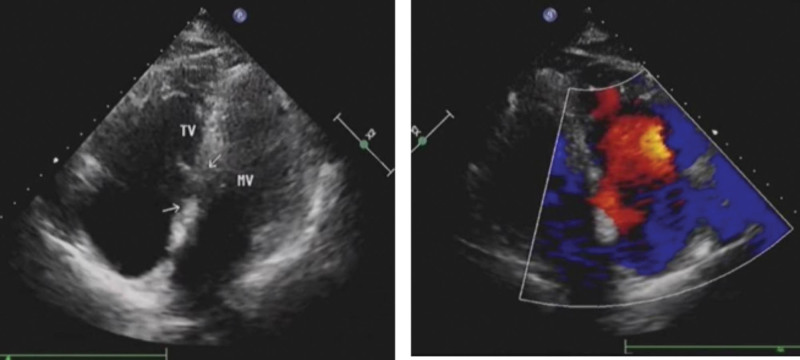
Admission echocardiogram of the patient. (A) Displacement of the tricuspid valve accompanied by atrial defect. (B) The position of Micra AV. (C) The position of AAI lead.

The patient, a 69-year-old female, was admitted to the hospital with bilateral lower limb edema, exertional dyspnea for 4 years, and intermittent syncope for 9 months. The electrocardiogram on admission showed sinus bradycardia, junctional escape rhythm, a heart rate of 44 bpm (Fig. 1A and B). A B-type natriuretic peptide (BNP) test yielded a result of 179 ng/mL (normal range: 0–100 pg/mL). Moreover, echocardiogram showed tricuspid valve displacement with severe insufficiency, atrial septal defect, right atrial enlargement, and 81% ejection fraction (Fig. 2). For patients with tricuspid valve displacement and severe tricuspid insufficiency, surgical treatment is recommended if symptoms develop. After thorough communication with cardiologists, tricuspid valve repair surgery was considered an option for the patient. However, the patient developed symptoms of dizziness, shortness of breath, and decreased activity tolerance. Electrocardiogram and Holter electrocardiogram indicated severe bradycardia, sinus arrest, and thus a definite diagnosis of SSS. Considering the patient’s condition and the indications of permanent pacemaker implantation, we decided to use a wired single-chamber atrial pacing + leadless ventricular pacing with atrial sensing, which could achieve atrioventricular synchronous pacing + leadless passage through the tricuspid valve. This pacing procedure would avoid exacerbating the increase in TR caused by the venous lead passing through the tricuspid valve and would not hinder subsequent surgical intervention for tricuspid valve disease.

**Figure 3.**
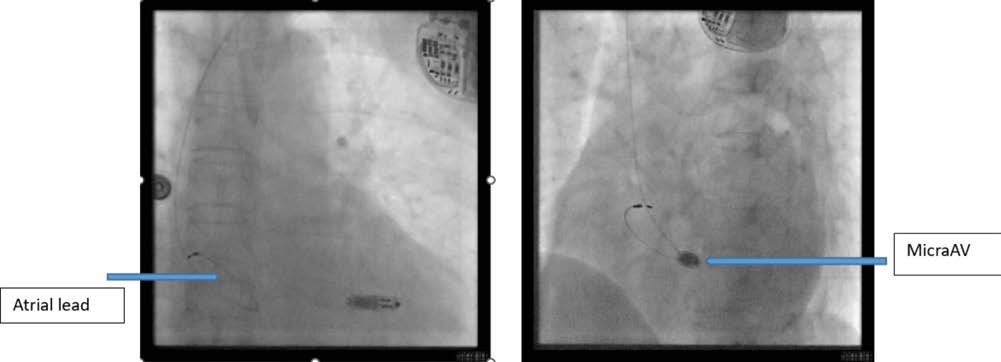
Postoperative imaging in the anteroposterior (AP) position and at 45° left anterior oblique (LAO) angle. Atrial pacing with an AAI lead and Micra AV was implanted in the right atrial septum and mid-right ventricular septum, respectively, with good pacemaker position.

**Figure 4.**
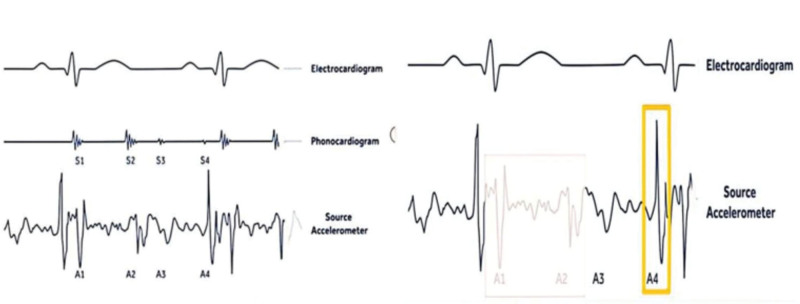
The Principle of MicraAV tracking atrial activity. A1: Start of ventricular systole, mitral and tricuspid valves close. A2: End of ventricular systole, aortic and pulmonic valves close. A3: Diastole passive blood flow from A to V, corresponds to E-wave on Doppler echo. A4: Atrial systole, blood pushed into ventricles 100 ms electromechanical delay, corresponds to A-wave on Doppler echo.

The patient consented to receive the pacemaker implantation. Implantation of a single-chamber pacemaker in AAI mode (single-chamber atrial pacing) (Model: EN1SR01 Ensure SR MRI) and implantation of leadless pacemaker (Model: Medtronic MedMC1AVR1; Medtronic, USA) were performed on December 29, 2022. The operation parameters of the AAI pacemaker were as follows: threshold 1.75 v/0.4 ms, sensing 2.0 mV, impedance 760 Ω. A diagonal incision 2 cm below the left clavicle was made, a pouch was created by blunt dissection, and then a temporary lead (Model: 3830; Medtronic, USA) was transvenously placed in the right ventricle. A right femoral vein puncture was performed, and the Micra delivery sheath was advanced through the right femoral vein to the inferior vena cava, then to the right atrium and through the tricuspid valve to the right ventricle. The sheath was used to implant the pacemaker into the mid-right ventricular septum, and Tug test showed good pacemaker position, as shown in Figure 3. Then the pacing parameters were tested: pacing threshold of 0.5 V, impedance of 600 Ω, P/R amplitude of 12 mV, and a pacing rate of 60 bpm. The Model 3080 lead was rotated back from the right ventricle and placed in the right atrial septum (Fig. 3). The Micra AV uses a 3-axis accelerometer to sense the contraction of the chamber and provides atrial synchoronous-ventricular inhibited (VDD) pacing mode. The accelerometer produces the corresponding A1 to A4 waveform to the heart sound S1 to S4. Post-ventricular atrial blank period (PVAB) is used to allow A1 and A2 signals to enter a blank period, so that these signals will not be tracked. When patients develop atrioventricular block, the A4 signal pacer will be tracked, and the first “perceived” signal after PVAB will be tracked, often maintaining a relatively high A3 sensitivity threshold to ensure that A3 will not be tracked (Fig. 4).

After the operation, the sheath was removed, the skin wound was sutured, and hemostasis was achieved by compression. Sandbag compression was applied, and the patient was returned to the ward. Postoperative electrocardiogram suggested an atrial pacing of 60 bpm and ventricular self-down transmission. Before discharge, the pacemaker programming was set to VDD mode, with a rate 50 of bpm, right ventricular sensing 13.8 mV, impedance 580 Ω, threshold 0.38 V, atrial pacing, and intrinsic ventricular conduction. The follow-up parameters after pacemaker surgery were as follows; Micra AV: pacing threshold 0.63 v/0.24 ms, sensing 6.9 mv, impedance 430 Ω, pacing requirement 6.0%; atrial electrode: threshold 1.75 v/0.4 ms, sensing 1.3 mv, impedance 532 Ω, pacing ratio of 86.5%. The patient had stable parameters and was in good general condition at 1 month and 3 month outpatient follow-ups after discharge. The patient has not undergone surgical treatment of the tricuspid valve at present.

## 3. Discussion

Atrial pacing has been shown to reduce the risk of atrial fibrillation and stroke in patients with sinus node dysfunction.^[5]^ Studies have shown^[6]^ that the annual incidence of second-degree or higher atrioventricular block in patients with SSS is about 0.6% to 1.8%. The risk of atrioventricular block is a little less definite in the single-chamber pacing mode with atrial pacing (AAI), which has been abandoned in clinical practice. Therefore, the safer dual-chamber pacemaker implantation should be considered, with atrial pacing as the main mode and ventricular pacing as a backup. Some studies have also found that the implantation of dual-chamber pacemakers can reduce the risk of pacemaker syndrome and improve quality of life.^[7]^

Pacemaker leads may cause TR through several mechanisms, including leaflet perforation, rupture of chordae tendineae or papillary muscles, or embedding of the lead between valve leaflets.^[8]^ Lead extraction is 1 of the measures to treat severe TR caused by pacemaker leads, but it requires high technical proficiency and carries significant risks. The reported incidence of traumatic TR is 5.6% to 15.1%.^[9]^ For patients with tricuspid valve displacement and severe tricuspid insufficiency, surgical treatment is recommended if symptoms develop,^[4]^ which is consistent with the opinion of the cardiac surgery consultation. However, the patient developed recurrent edema of lower extremity and, abdominal distension prior to pacemaker implantation and dizziness, shortness of breath, and activity tolerance prior to admission. Combined with the patient’s electrocardiogram performance, the physician estimated that the symptoms of dizziness, shortness of breath, and reduced activity tolerance would be alleviated after correcting the slow heart rate. The patient demonstrated sufficient indications for pacemaker implantation, and the improvement in symptoms of dizziness, shortness of breath, and reduced activity tolerance further supported this decision. The patient and her families had a positive attitude towards pacemaker implantation but were relatively cautious about surgical intervention for the tricuspid valve. However, the option for future surgical intervention will continue to be considered and preserved.

Based on echocardiography, BNP test, and clinical symptoms, the case in this study was diagnosed with tricuspid valve displacement with severe insufficiency, significant enlargement of the right heart, and right heart failure. Ventricular pacing leads passing through the tricuspid valve may exacerbate tricuspid insufficiency and hinder subsequent surgical treatment of tricuspid valve disease. Epicardial pacing leads can only be placed during cardiac surgery, and pacing through a lead implanted in the left ventricle via the coronary vein is epicardial pacing with a low degree of myocardial synchronization. Considering all these factors, the preferred ventricular pacing method for this patient is leadless endocardial pacing in the right ventricle. His-bundle pacing involves the transmission of electrical signals through the His-Purkinje system, achieving physiological electromechanical synchronization. Additionally, the pacing lead can be fixed on the atrial side of the tricuspid-septal valve, without interfering with tricuspid valve closure. However, the long-term effectiveness and safety of this leadless pacing mode remain to be confirmed by large-scale prospective clinical studies. Our patient had SSS and was dependent on atrial pacing. Although Micra AV can provide VDD pacing, atrial pacing cannot be offered. Our solution was to connect the atrial lead to a single-chamber pulse generator to provide AAI pacing. Micra AV, via a 3-axis (*x*, *y*, *z*) accelerometer, can not only provide ventricular pacing, but also sense the activity of atrial contraction to achieve atrioventricular synchronization. The algorithm of Micra AV can sense the acceleration signal caused by the mechanical movement of the heart. Thus, Micra AV can track atrial waves and provide VDD pacing mode. It has been reported that a dual-pacemaker strategy with a single-chamber atrial pacemaker and a VDD leadless pacemaker can successfully provide atrioventricular sequential pacing and can feasibly reduce the degree of lead-induced TR.^[10]^ The patient had stable parameters and was in good general condition at 1 month and 3 month outpatient follow-ups after discharge.

The patient’s current state of coexistence of the 2 devices is temporary. Her attitude toward the subsequent intervention of the tricuspid valve is still unclear, but the options for the management of the tricuspid valve will be clarified at the end of the useful life of both devices (around 10 years). At that time, the devices will be replaced with 1 device. If only 1 device is chosen without compromising subsequent tricuspid valve interventions, the only choices are Micra or AAI pacing. Unfortunately, although atrial pacing in the AAI mode is sufficiently physiologic, it is not safe enough to pace the atrium alone without ventricular pacing because of the possibility of subsequent high-grade atrioventricular block. VVI may result in a loss of atrial synchronization, and also exacerbate the deterioration of cardiac function. Therefore, both the physicians and the patient’s family agreed that it was more important to choose the optimal procedure for the patient without significantly increasing cost-effectiveness.

Right ventricular leadless pacing was chosen so as not to interfere with possible future tricuspid valve surgery, rather than assuming that right ventricular leadless pacing must have less effect on tricuspid valve insufficiency than a wired pacing. The authors’ hospital has had several similarly troublesome cases requiring tricuspid valve surgery in the presence of the lead crossing the valve. For example, a patient who had a previously implanted pacemaker with pacing lead was surgically treated for severe tricuspid insufficiency. During the surgery, the cardiac surgeon cut the pacing lead and removed the intracardiac lead, and implanted epicardial temporary pacing lead. After the tricuspid valve surgery, the cardiologist removed the permanent pacing lead that remained in the superior vena cava through the subclavian vein route. Reimplantation of a right ventricular pacemaker with leads or a leadless pacemaker was then conducted depending on the conditions of the patient during and after the tricuspid valve surgery. intraoperative and postoperative conditions of the patient’s valve surgery. There is always some conflict between the presence of the leads crossing the tricuspid valve and the tricuspid valve surgery. Thus, we believe that a leadless ventricular pacemaker is the preferred option for our case.

A secondary argument for choosing leadless pacing is that we found that the lead across the tricuspid valve can lead to tricuspid valve abnormalities for the following 2 reasons. First, long-term friction between the lead and the tricuspid valve and its attachments can easily lead to adhesion between the lead and the tricuspid valve and its attachments as well as chronic inflammation, consequently affecting the function of the tricuspid valve. This has been confirmed in some cases during cardiac surgery. In addition, even if the lead reservation is adequate, the length of the right ventricular lead is sufficient in many patients when the pacemaker is replaced many years later. We found that the effect of a taut right ventricular lead on tricuspid valve closure was severe with ultrasound imaging. The above situation caused by a wired pacemaker can be avoided with right ventricular leadless pacing.

Patients who require atrial pacing due to sinus node dysfunction can also consider a wired single-chamber atrial pacing + leadless ventricular pacing if the tricuspid valve is not suitable for implantation of a leaded pacemaker. However, this pacing procedure is limited to the current situation where the leadless pacemaker is not able to efficiently pace the atrium. This technique will not be necessary if the leadless pacemaker can function as an atrial pacemaker and can produce effective electrical communication with ventricular pacing. In our case with EA and SSS, both leaded and leadless pacemakers were implanted without any problems; the implantation of the electrodes or the preparation of a pouch were accomplished successfully. Moreover, the 2 pacemakers did not interfere with each other as observed through postoperative follow-up. The programing head may detect both devices during the postoperative follow-up using a pacemaker programmer, and different devices can be selected on the display to monitor and question.

The novel atrioventricular sequential pacing method using a transvenous atrial pacemaker and Micra AV reserves surgical space for valve replacement in patients with valvular disease that requires surgical treatment, and also provides a safe, physiological pacing method. However, the long-term safety and efficacy of this surgical approach need to be observed.

## Author contributions

**Conceptualization:** Yongxiang Cai.

**Data curation:** Yongxiang Cai, Mingjiang Liu, Yan Xiong.

**Formal analysis:** Mingjiang Liu, Jianhong Tao, Yijia Tang.

**Methodology:** Yongxiang Cai, Jianhong Tao, Yijia Tang.

**Writing – original draft:** Yan Xiong.

**Writing – review & editing:** Jianhong Tao, Yijia Tang, Yan Xiong.
